# The Terrible Triad of the Elbow Accompanied by Capitellum and Humerus Shaft Fracture: A Rare Case Report

**DOI:** 10.7759/cureus.27658

**Published:** 2022-08-03

**Authors:** Tolgahan Cengiz, Şafak Aydın, Furkan Erdoğan, Hamisi M Mraja, Hüseyin Sina Coşkun

**Affiliations:** 1 Orthopaedics and Traumatology, Samsun Ondokuz Mayıs University Faculty of Medicine, Samsun, TUR; 2 Orthopaedics and Traumatology, Sabuncuoğlu Şerefeddin Research and Training Hospital, Amasya, TUR; 3 Orthopaedics and Traumatology, Istanbul Spine Center, Istanbul Florence Nightingale Hospital, Istanbul, TUR

**Keywords:** humerus shaft, fluoroscopy, capitellum, elbow dislocation, elbow fractures, terrible triad

## Abstract

The terrible triad of the elbow consists of radial head fracture and coronoid process fracture in addition to posterior dislocation of the elbow. It indicates high-risk complications such as instability, malunion, nonunion, and proximal radioulnar synostosis. We describe a rare case that was admitted to the emergency service with a terrible triad of the elbow with additional capitellum fracture, lateral collateral ligament (LCL) injury, and ipsilateral humeral shaft fracture. We treated the patient urgently by performing osteosynthesis of the humeral shaft fracture, radial head fracture, coronoid fracture, capitellum fractures, and repair of the LCL rupture. The terrible triad of the elbow also can be accompanied by adjacent column fractures, including the humeral shaft. In such complex cases, preoperative planning should be done well, and the entire anatomy should be demonstrated with additional imaging. Optimal treatment of all the fractured bones and ligaments is critical for early rehabilitation. The main aim of surgery is to acquire desired results by starting an early rehabilitation, including joint movement.

## Introduction

Hotchkiss described the terrible triad of the elbow in 1996. The definition consists of radial head fracture, coronoid process fracture, and posterior dislocation of the elbow. The terrible triad terminology indicates high-risk complications such as instability, malunion, nonunion, and proximal radioulnar synostosis. The mechanism of injury is usually seen in extended elbows exposed to valgus stress trauma [1]. Axial loading from the radius along the distal humerus can cause a shear fracture of the capitellum [2]. Shear fracture of the capitellum is extremely rare, accounting for less than 1% of all elbow fractures [3,4].

In this case report, we aim to present a rare case admitted to the emergency service with a terrible triad of the elbow accompanied by capitellum fracture, lateral collateral ligament (LCL) injury, and ipsilateral humeral shaft fracture.

## Case presentation

Physical examination and radiological studies

**Figure 1:**
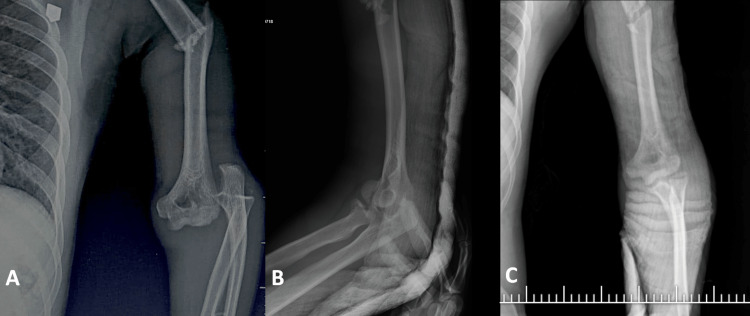
(A) X-ray of the left arm demonstrating humerus shaft fracture with an ipsilateral elbow dislocation. (B and C) Lateral X-ray view of the left arm demonstrating reduced elbow dislocation with a clear demonstration of the radial head and capitellum fractures.

A 23-year-old male patient was admitted to our emergency department. He had a history of a fall from a height on his left upper extremity. The left arm and elbow had a deformed appearance on the physical examination. Moderate swelling of the arm was detected. No visible wounds were seen on the skin. There was a pathological movement on the left arm and a restricted range of motion on the left elbow. Vascular and neurological examination of the patient’s left upper extremity was intact. Emergency radiological evaluation showed a humerus shaft fracture with an ipsilateral dislocated elbow (Figure 1). An emergency closed reduction was performed in the emergency department (Figure 1). After elbow reduction, repeated elbow examinations were performed, and instability was detected. For further evaluations, a computed tomography (CT) scan was performed after closed reduction. Ipsilateral humeral shaft, ipsilateral capitellum, ipsilateral radial head, and ipsilateral coronoid fractures were detected. There was no additional trauma in the advanced physical examination of the patient.

Surgical procedure and outcome

**Figure 2:**
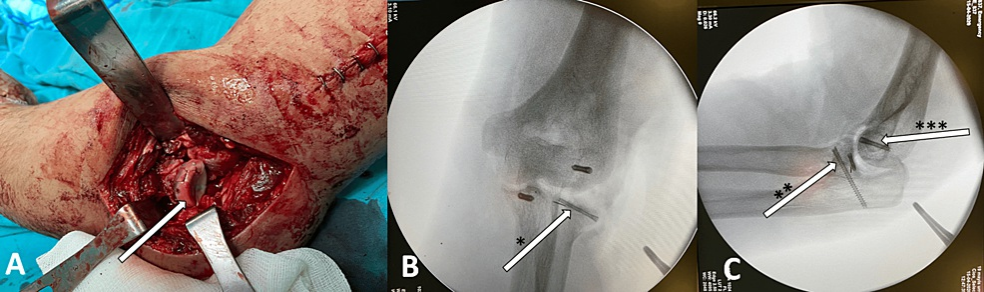
Intraoperative views: (A) Kocher approach demonstrating osteosynthesis of radial head fracture with a headless screw. (B) Fluoroscopic AP view of the elbow. (*) Arrow demonstrating radial head fracture osteosynthesis with a headless screw. (C) Fluoroscopic lateral view of the elbow. (**) Arrow demonstrating coronoid fracture osteosynthesis with a headless cannulated screw. (***) Arrow demonstrating capitellum fracture osteosynthesis with a headless cannulated screw.

Immediately after preoperative evaluations, the patient was operated on urgently. Firstly, plate-screw osteosynthesis was performed with a lateral approach for the humeral shaft fracture. In this approach, the radial nerve was observed as intact. Throughout the surgery, the nerve was carefully protected. In addition, it was observed that the LCL ligament was partially ruptured through a lateral incision in the left elbow with the Kocher approach. The radial head fracture was reduced and fixed with a 2.7-mm headless screw (Figure 2A, 2B). Subsequently, coronoid fracture osteosynthesis was achieved using a headless cannulated screw through an anterior incision of the elbow (Figure 2C). The elbow was reduced, and the capitellum was fixed with a headless cannulated screw (Figure 2C). The LCL rupture was repaired with an anchor suture. The procedure was terminated after the elbow joint was declared stable in the intraoperative examination under fluoroscopy. On the postoperative second day, a wrist drop developed. The posterior interosseous nerve (PIN), a motor branch of the radial nerve, was considered for neuropraxia. Close follow-up was agreed upon.

**Figure 3:**
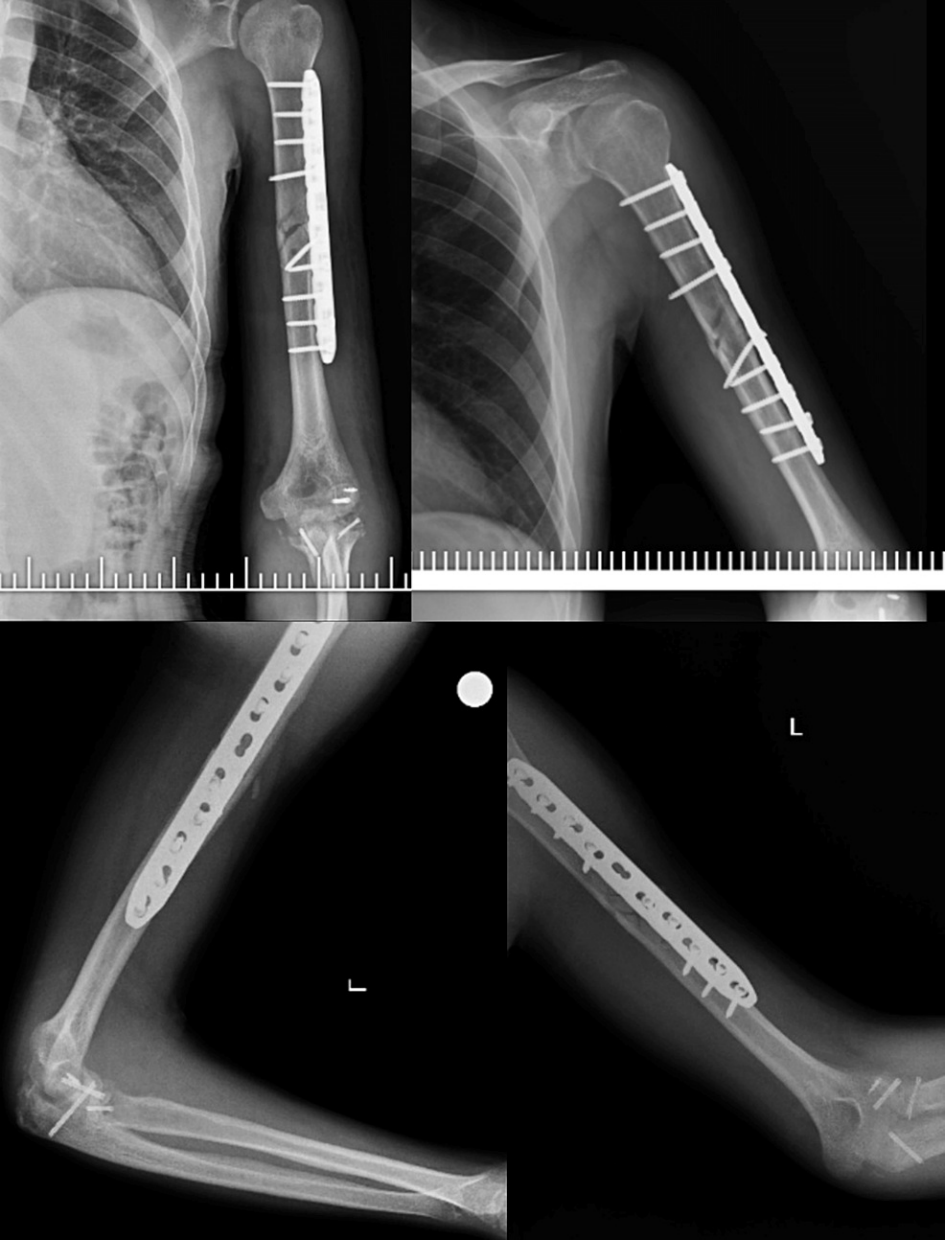
Late follow-up X-ray views demonstrating osteosynthesis of humerus shaft, capitellum, coronoid, and radial head fractures.

In the postoperative first month examination, the nerve damage had healed without any sequelae. At this period, a postoperative radiological evaluation was made, and callus was considered enough in the X-rays (Figure 3).

In the postoperative sixth month examination, the patient could move his elbow from 0° to 120° of flexion, full supination, and pronation.

## Discussion

The elbow joint consists of four major columns: anteriorly, the brachialis muscle, anterior capsule, and coronoid process; medially, the medial collateral ligament, medial condyle, and coronoid process; laterally, the LCL, lateral condyle, and capitellum; and posteriorly, the olecranon, triceps muscle, and posterior capsule [5]. Each structure in these four columns is essential for maintaining the joint’s stability and range of motion.

Elbow dislocations are categorized into two groups: simple and complex. A simple dislocation of the elbow is a capsuloligamentous injury without fracture. A complex elbow dislocation is associated with bone injuries. Complex elbow dislocations, which are accompanied by radial head and coronoid process fractures, have been named the “terrible triad” by Hotchkiss due to their poor posttreatment outcomes [6].

The treatment of the terrible triad of the elbow is challenging. Also, the complications that develop after the surgery are as difficult to treat. The most known complications of terrible triad injuries include redislocation, residual instability, elbow stiffness, heterotopic ossification, olecranon bursitis, fracture malunion or nonunion, elbow arthrosis, and ulnar or radial nerve palsy [7]. Recurrent dislocation or subluxation is the most challenging complication [8].

Although the treatment of the terrible triad of the elbow is challenging, the accompanying capitellum fracture and humeral shaft fracture in our case make the treatment more challenging. Although cases of terrible triad accompanied by capitellum fractures have been reported in the literature review, no case with ipsilateral humeral shaft fracture accompanying these four pathologies has been reported [1,5].

Advanced imaging is required for evaluating the complex anatomy of the elbow joint. In this case, besides X-ray evaluations, computed tomography (CT) scan was critical in the preoperative planning. A better study of the entire anatomy and bone fractures with CT after reduction is an essential step in the operation plan [9]. Humerus shaft, capitellum, coronoid, and radial head fractures were detected by X-ray and CT. Surgical planning was done by revealing the entire anatomy.

The surgical approach is decided in elbow injury cases after cautious planning preoperatively. Medial and lateral approaches have limitations in fractures involving the trochlea, capitellum, and anterior articular surface of the coronoid. Coronoid fractures are difficult to visualize when using a lateral approach, especially when the radial head is intact. The medial approach requires the dissection of the ulnar nerve, flexor muscle, and pronator muscle. In the posterior approach, olecranon osteotomy is often mandatory. The anterior approach provides a safe and reproducible way into the entire anterior joint surface of the elbow [10].

In our case, we performed three different approaches. First, a lateral incision was used to stabilize the humeral shaft fracture. Then, a Kocher approach was used to perform osteosynthesis of the radial head fracture using a 2.7-mm headless screw. Finally, an anterior approach was used to fix the coronoid fracture with a screw, reduce the elbow, and ultimately repair the LCL tear with an anchor suture. The case was terminated after ensuring the elbow’s stabilization by examination under fluoroscopy.

The postoperative rehabilitation process is as challenging as the surgical procedure in such complex cases. It is essential to start joint motion early after ensuring that the elbow is stabilized. Elbow immobilization for more than four weeks has been associated with poor clinical outcomes [2]. A long arm splint extending to the shoulder was applied to the patient for two weeks postoperatively. Later, we started mobilization. Afterward, a functional brace was applied, and elbow joint movements were allowed. In the postoperative sixth month, bone union was detected radiographically. Also, the elbow joint’s ability to extend 0° of extension, 120° of flexion, and full supination-pronation were pleasing.

Although, in this case, the combination of anterior and lateral incisions protected the posterior interosseous nerve (PIN) branch of the radial nerve, loss of wrist and thumb extension were observed in the early postoperative period. Early nerve examination without any deficit and the development of nerve deficit on the second day is due to hematoma causing nerve compression. In these cases, as long as intraoperative nerve protection was performed, we evaluate them as neuropraxia of the PIN branch and should be closely monitored. Accordingly, wrist movements were regained with the regression of our patient’s swelling and edema around the elbow.

## Conclusions

The terrible triad of the elbow is challenging in terms of treatment and rehabilitation. In addition, humerus shaft and capitellum fractures accompanied by these cases make the treatment challenging. However, it is possible to achieve the desired results by starting an early rehabilitation program including joint movements. In such complex cases, preoperative planning should be done comprehensively. Advanced imaging studies can be included to evaluate the complex anatomy. Moreover, complications related to the soft tissue (vascular and nerves) may be present in elbow fractures, especially in cases associated with elbow dislocation.

Combined approach usage to the elbow joint will reduce nerve injuries and allow excellent osteosynthesis. Furthermore, postoperative neuropraxia of the radial nerve must be carefully evaluated. Even with cautious nerve protection intraoperatively, neuropraxia can develop after hematoma accumulation around the elbow.
